# Use of Biofeedback-Based Virtual Reality in Pediatric Perioperative and Postoperative Settings: Observational Study

**DOI:** 10.2196/48959

**Published:** 2024-09-16

**Authors:** Zandantsetseg Orgil, Anitra Karthic, Nora F Bell, Lisa M Heisterberg, Sara E Williams, Lili Ding, Susmita Kashikar-Zuck, Christopher D King, Vanessa A Olbrecht

**Affiliations:** 1 Abigail Wexner Research Institute Nationwide Children's Hospital Columbus, OH United States; 2 Department of Anesthesiology The Ohio State University College of Medicine Columbus, OH United States; 3 Department of Anesthesiology & Pain Medicine Nationwide Children's Hospital Columbus, OH United States; 4 Department of Pediatrics University of Cincinnati College of Medicine Cincinnati, OH United States; 5 Department of Anesthesiology, Perioperative and Pain Medicine Stanford University School of Medicine Palo Alto, CA United States; 6 Division of Biostatistics and Epidemiology Cincinnati Children’s Hospital Medical Center Cincinnati, OH United States; 7 Pediatric Pain Research Center Division of Behavioral Medicine and Clinical Psychology Cincinnati Children's Hospital Medical Center Cincinnati, OH United States; 8 Department of Anesthesiology and Perioperative Medicine Nemours Children’s Health, Delaware Valley Wilmington, DE United States

**Keywords:** virtual reality, biofeedback, biofeedback-based virtual reality, acute pain, postoperative pain, pediatrics, postoperative, pain, anxiety, children, adolescents, perioperative management, acceptability, feasibility, pain reduction

## Abstract

**Background:**

Biofeedback-based virtual reality (VR-BF) is a novel, nonpharmacologic method for teaching patients how to control their breathing, which in turn increases heart rate variability (HRV) and may reduce pain. Unlike traditional forms of biofeedback, VR-BF is delivered through a gamified virtual reality environment, increasing the accessibility of biofeedback. This is the first study to systematically integrate VR-BF use in the pediatric perioperative setting, with the ultimate goal of evaluating the efficacy of VR-BF to reduce pain, anxiety, and opioid consumption once feasibility and acceptability have been established.

**Objectives:**

The primary objective was to develop a clinical trial protocol for VR-BF use in the pediatric perioperative setting, including preoperative education and training, and postoperative application of VR-BF in children undergoing surgery. A secondary objective was to evaluate the patient and parent experience with VR-BF.

**Methods:**

A total of 23 patients (12-18 years of age) scheduled for surgery at Nationwide Children’s Hospital were recruited using purposive sampling. Following training, participants independently completed a daily, 10-minute VR-BF session for 7 days before surgery and during their inpatient stay. Participants could use VR-BF up to 2 weeks after hospital discharge. Patient- and session-level data of VR-BF usage and achievement of target HRV parameters were measured to identify the optimal frequency and duration of sessions before and after surgery for this population. Standardized questionnaires and semistructured interviews were conducted to obtain qualitative information about patients’ experiences with VR-BF.

**Results:**

Patient-level data indicated that the highest odds of achieving 1 session under target HRV parameters was after 4 sessions (odds ratio [OR] 5.1 for 4 vs 3 sessions, 95% CI 1.3-20.6; OR 16.6 for 3 vs 2 sessions, 95% CI 1.2-217.0). Session-level data showed that a session duration of 9 to 10 minutes provided the greatest odds of achieving 1 session under target HRV parameters (OR 1.3 for 9 vs 8 min, 95% CI 1.1-1.7; OR 1.4 for 8 vs 7 min, 95% CI 1.1-1.8; OR 1 for 10 vs 9 min, 95% CI 0.9-1.2). Qualitative data revealed patient satisfaction with the VR-BF technology, particularly in managing perioperative stress (17/20, 85%). Few patients reported VR-BF as beneficial for pain (8/20, 40%).

**Conclusions:**

Children and adolescents undergoing surgery successfully learned behavioral strategies with VR-BF with 10-minute sessions once daily for 5 days. To integrate VR-BF as a therapeutic intervention in a subsequent clinical trial, patients will be instructed to complete three 10-minute sessions a day for 7 days after surgery.

**Trial Registration:**

ClinicalTrials NCT04943874; https://clinicaltrials.gov/ct2/show/NCT04943874

## Introduction

For many patients, the postoperative period is associated with significant and sometimes uncontrolled pain [1-4]. Not only can these circumstances lead to higher morbidity, increased hospital costs, and longer recovery times, but uncontrolled postoperative pain also increases the risk of exposure to and persistent use of opioids [4-8]. Despite greater emphasis on the use of multimodal, opioid-sparing analgesic regimens for postoperative pain, the percentage of patients experiencing severe pain after surgery has not changed significantly since the early 2000s, and narcotics remain the primary treatment for pain management [9-11]. Thus, the demand for nonpharmacologic alternative therapies for pain control has never been greater for children and adolescents [12].

One nonpharmacologic alternative is biofeedback, a mind-body therapy that provides sustained pain relief [13,14] in various clinical settings [15-26]. Biofeedback reduces pain by teaching patients behavioral modifications (eg, decreasing respiratory rate) to change their physiological status (eg, increasing heart rate variability [HRV]) [27], characterized as the increase and subsequent decrease in heartbeats during inhalation and exhalation, respectively [28]. Higher HRV downregulates the sympathetic nervous system and activates the parasympathetic nervous system, increasing vagal tone and reducing pain [29,30]. However, many barriers exist to the routine use of biofeedback [31], including the need for trained personnel and specialized equipment, and the lack of patient engagement and motivation for session repetition [32]. Thus, alternative strategies to deliver this effective therapy at point-of-care are needed in children and adolescents.

As technological advances have allowed for greater use of virtual reality (VR), VR has been implemented in many clinical situations to minimize pain during acutely painful procedures [33-42]. The sense of immersion created by VR can complement the therapeutic effects of distraction therapy during short, painful procedures by redirecting attention [43,44] and engaging the patient in simple mind-body therapies such as guided relaxation and slow breathing [45,46]. However, to date, VR-based delivery of distraction- and relaxation-based therapies have shown only transient reductions in pain that are insufficient to assist with more prolonged pain experiences, including postoperative pain [45,47-49].

To fill the unmet critical need for accessible, nonpharmacologic analgesia, we are exploring the integration of biofeedback with VR (VR-BF) as a promising new therapy that may be effective for postoperative pain management and may overcome the challenges of existing mind-body interventions [50]. However, VR-BF has yet to be systematically used in the perioperative period; thus, no defined treatment protocols exist for its application [51]. This study aimed to refine a treatment protocol for preoperative education and training and postoperative application of VR-BF in children and adolescents undergoing surgery requiring management by the Acute Pain Service by assessing the impact of VR-BF use on HRV parameters. To gain additional qualitative acceptability data of this technology, standardized questionnaires were used to assess patient and parent perceptions of their experience with VR-BF.

## Methods

### Overview

This single-center, prospective observational study of pediatric surgical patients aimed to refine a VR-BF protocol consisting of a preoperative education and training period to identify the optimal frequency and duration of VR-BF sessions to achieve target physiological parameters. Findings from this study will inform the design of a clinical trial to assess the ability of a VR-BF intervention to reduce postoperative pain, anxiety, and opioid consumption in children and adolescents.

### Ethical Considerations

This study was approved by the institutional review board (#STUDY00002080) at Nationwide Children’s Hospital (NCH) and conducted per the rules and regulations for ethical clinical research. This study was registered with ClinicalTrials.gov on May 17, 2021 (NCT04943874) and adhered to the CONSORT (Consolidated Standards of Reporting Trials) guidelines. Written consents from parents (and assent for patients younger than 12 years) were obtained from all participants before the first study visit. A stipend of up to US $100 per patient was given for completing all pre- and postsurgical study procedures.

### Patients

A total of 23 patients scheduled for surgery anticipated to cause moderate to severe pain were recruited using purposive sampling between March 2022 and September 2022. Patients at NCH undergoing surgical procedures associated with moderate to severe pain (eg, laparotomy and spine surgery) are managed by the Acute Pain Service and receive intravenous opioids for pain management. All patients received standard postoperative care and were not withheld from medications during study participation.

Patients were identified up to 2 months in advance of their surgery for recruitment. Eligibility criteria can be found in Textbox 1.

**Inclusion and exclusion criteria.**

**Inclusion criteria**

- 12-18 years old (all inclusive)
- Able to read, understand, and speak English
- Scheduled to undergo surgery at Nationwide Children’s Hospital anticipated to cause moderate to severe pain with 1-night postoperative hospital admission
- Require postoperative pain management by the Acute Pain Service
- Own or have access to a mobile device or computer

**Exclusion criteria**

- Younger than 12 years or older than 18 years
- Non–English-speaking
- History of significant developmental delay, psychiatric conditions associated with hallucinations or delusions, or significant neurological disease, especially epilepsy or seizure disorder
- History of significant motion sickness
- History of chronic pain
- Chronically using opioids or benzodiazepines for the management of pain preoperatively
- Actively experiencing nausea or vomiting
- Any conditions that preclude their ability to use the VR headset, such as craniofacial deformities or surgeries of the head and neck

### Equipment

**Figure 1:**
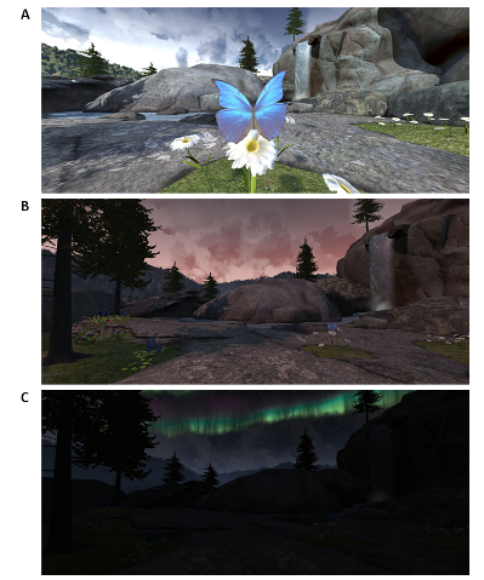
Snapshots of the Mindfulness Aurora app. (A) Daytime scene as patients are verbally instructed to sync their breathing with the wings of a floating butterfly. (B) The Mindfulness Aurora app transitioning from day to night. (C) Night scene in the Mindfulness Aurora app in which patients are verbally instructed to sync their breathing with the Northern Lights.

All participants in this study used the Meta Quest2 VR headset (Meta Platforms Inc) and the guided relaxation-based VR app, Mindfulness Aurora, developed by the Stanford Chariot program. Mindfulness Aurora encourages relaxation practice by being focused on slow breathing. Patients are transported to an alpine meadow, where visual and auditory cues associated with the changing environment prompt participants to mirror and synchronize their breathing to the app. These changes include floating butterflies, swaying trees, and cloud movements as the 3-dimensional world transitions from day to night over a period of 10 minutes (Figure 1).

Patient physiological parameters were recorded during VR-BF sessions using HeartMath Inner Balance (HeartMath Institute), a commercially available heart rhythm monitoring device used to teach patients biofeedback. HRV is collected using an ear-clip sensor and integrated directly with the Inner Balance mobile app through Bluetooth. Data were then stored in an online data cloud accessible by the study team using HeartMath’s emWave Pro software. HeartMath uses a method of quantifying heart rhythms derived from spectral power analysis, and the ideal fluctuations in the HRV waveform over time are depicted as a sine wave on a power spectrum [28]. The degree of how sine-wave-like the user’s HRV pattern was scored into low (poor), medium, and high (good) states of coherence. Each coherence state is then assigned a numerical value depicting the proportion of time the user was in each state of low, medium, and high coherence (displayed as red, blue, and green, respectively, in emWave Pro).

### Biofeedback-Based VR Sessions

#### Before Arrival for Surgery

Participants underwent a single in-person or virtual training session, up to 2 weeks before surgery. During this visit, participants watched a prerecorded video on the benefits of HRV biofeedback and received a scripted device and content tutorial from a trained clinical research coordinator. Participants were instructed to independently complete a daily, 10-minute session at home for 7 days before their procedure. Session frequency and duration were determined in line with the standard protocol for mind-body therapies [29,52]. Participants were asked to record each session (date, time, and duration) directly into a web-based data capture tool, REDCap (Research Electronic Data Capture; Vanderbilt University).

#### Day of Surgery and Post Surgery

Participants were instructed to bring the devices to the hospital on the day of surgery and to resume daily sessions for the duration of hospital admission starting on postoperative day 0. Patients also had the opportunity to use VR-BF as needed outside of the daily 10-minute session while admitted to the hospital, particularly when in pain. A study team member provided technical assistance as needed.

A final study visit was conducted before discharge to obtain patient feedback using investigator-derived questionnaires. For those who opted to continue independent pain management with VR-BF for up to 14 additional days after hospital discharge, the final study visit was scheduled after the additional time or when participants decided to stop, whichever came first. The same web-based data capture tool was used for recording postoperative sessions. The final study visit occurred in person (in the clinical research department or at a surgery follow-up visit) or by phone.

### Measures

The primary outcome of this study was the development of a VR-BF treatment protocol, including the frequency and duration of sessions before and after surgery for children and adolescents to be applied in a future efficacy trial.

#### Patient Information

Before surgery, the patient’s age, sex, race, ethnicity, comorbidities, and current pain or anxiety medications were collected. Following surgery, the patient’s American Society of Anesthesiologists (ASA) status [53], diagnosis at surgery, surgery type, and anesthesia type and duration were also collected.

#### Biofeedback-Based VR and Heart Rate Variability

The patient’s ability to complete at least 1 session in which 50% or more of the session time achieved high HRV coherence (target parameter) was recorded. Changes in the frequency (number of sessions) and duration (time in minutes) of VR-BF sessions completed during the preoperative and postoperative periods were measured. The target parameter was selected based on the clinical experience of what constitutes a relaxing breathing training session for youth and youth with pain using the metrics available through the HeartMath program indicating success (eg, achieving “green” when in high HRV coherence).

#### Patient Experience

Patient experience was measured with a questionnaire (patient experience questionnaire—child [PEQ-C]; Multimedia Appendix 1) created by the study investigators. A similar survey (patient experience questionnaire—parent [PEQ-P]; (Multimedia Appendix 2) was given to the participant’s parent or guardian to understand their experience and perspective with their child using VR-BF; PEQ-P was optional for parents of adult patients. Patients and parents used a 5-point Likert scale to rank the extent to which they agree to statements on the PEQ-C and PEQ-P from “strongly agree” to “strongly disagree.” Responses to each questionnaire item provided feedback for making iterative protocol refinements on 5 domains of VR-BF acceptability, that are VR content and usability, preoperative education and training, postoperative application, perceived efficacy, and acceptability and satisfaction.

Participants completed surveys at the final study visit on paper or electronically with an iPad (Apple, Inc).

### Statistical Analysis

#### Overview

Statistical analysis was conducted using SAS 9.4 (SAS Institute). A *P* value of .05 was the cutoff for statistical significance. Although no confirmatory hypothesis testing was done, exploratory analyses were conducted to investigate the association, if any, between VR-BF and HRV parameters. Any missing data were examined, and all available data were used in the statistical analyses.

#### Descriptive Analysis

Descriptive statistics (categorical variables: frequency and percentage; continuous variables: mean and SD or median and IQR) were generated for study variables, including baseline characteristics and perioperative VR-BF use (adjusted and nonadjusted for preoperative, postoperative, and home sessions).

#### Associations Between Biofeedback-Based VR Use and Achievement of Target Heart Rate Variability

Logistic and spline regression with and without adjustment for preoperative, postoperative, or home VR-BF applications were used to explore relationships between different frequencies and durations of VR-BF sessions and patients’ ability to achieve a high HRV coherence for 50% or more of a session time. Patient-level and session-level data were used to determine the appropriate VR-BF dosing and to refine the treatment protocol.

#### Analysis of Patient-Level Data

A total of 2 outcomes were separately derived at the patient level by preoperative, postoperative, or home use. One is the percentage of sessions achieving target parameters: n_1_/n, where n_1_ is the number of sessions achieving target parameters, and n is the total number of sessions. The other is a binary outcome of any session achieving the target parameter, which equals 1 if n_1_>0, 0 otherwise.

Patient-level number of sessions and average session duration were also derived separately by preoperative, postoperative, or home use. Comparisons in the number of sessions and average session duration between patients completing at least 1 session with 50% or more of session time under target HRV coherence versus those that did not (miss) were conducted using Wilcoxon rank-sum tests. Nonlinear regression and nonlinear logistic regression with spline (for the number of sessions and average session duration) were used for the 2 outcomes, the percentage of sessions, and any session achieving the target HRV parameter, respectively. This allowed us to examine the impact of frequency and duration of VR-BF use on the outcomes while adjusting for preoperative, postoperative, or home use. Random participant effect was included in the models when significant.

#### Analysis of Session-Level Data

A binary outcome of a session achieving or failing to achieve the target HRV parameter was derived at the session level. Its association with session number and duration was examined using logistic regression with spline for session-level outcomes while adjusting for preoperative, postoperative, or home use. Random participant effect was included in the models when significant.

### Sample Size

Due to the nature of this pilot study, no statistical power analysis was done to determine the sample size. Instead, the sample size was based on findings from this team’s work in a previous pilot clinical trial [45,49,54,55], the investigators’ clinical experiences with the patient population, and existing literature on protocol refinements in intervention development [56,57]. Purposive sampling was used for a representative patient population. Patient enrollment and data review were carried out in groups of 4 to allow for iterative protocol refinements between patients.

## Results

### Participant Characteristics

Over 8 months, 23 patients were enrolled in this study. Data from 22 (96%) patients were included in the final analysis; 1 (4%) dropped out on the second day of study participation. The education and training session was conducted preoperatively for 22 (96%) participants, and 1 participant completed the education and training session on postoperative day 1. Although this patient deviated from protocol due to noncompliance with the study protocol, the patient’s data are included in the analysis as data were obtained. Missing data resulted from challenges in patient adherence, including compliance to study protocol, experiencing pain and other negative symptoms due to surgery, and inability to contact the patients’ families.

Most participants were female (16/23, 70%) and Caucasian (19/23, 83%), consistent with the demographics of our surgical population (Table 1). Of the 23 patients, 9 (39%) underwent abdominal, bariatric, colorectal, or urological surgeries; 2 (9%) underwent chest procedures; and 12 (52%) underwent orthopedic surgery. Most patients (14/23, 61%) were classified as ASA physical status I or II, and 39% (9/23) were classified as ASA physical status III or IV (Table 1).

**Figure 2:**
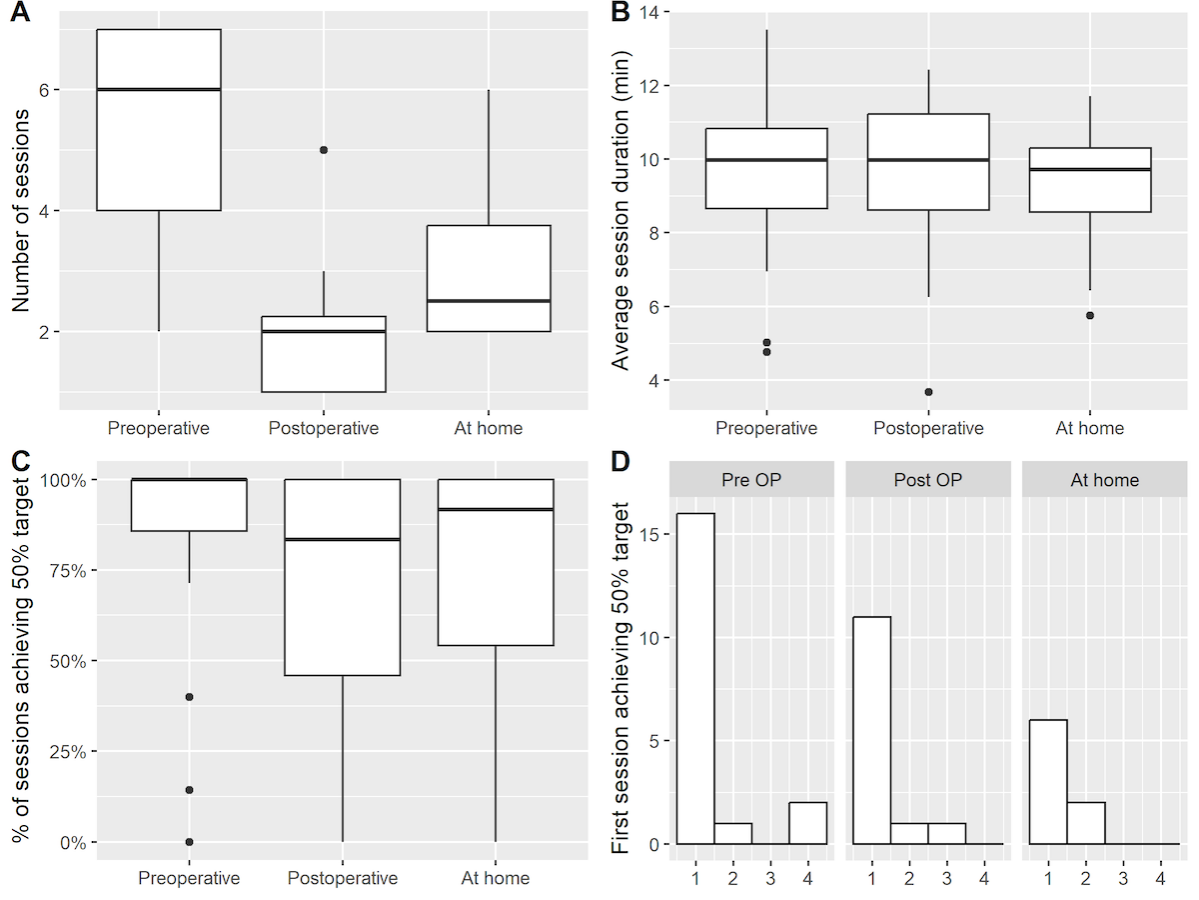
Boxplots of (A) number of sessions, (B) average session duration, (C) percentage of session achieving target, and (D) histogram of the first session number to achieve target using patient-level data.

In the preoperative period, 87% (20/23) out of the total number of enrolled patients completed ≥1 session (median 6, IQR 4-7; Figure 2) with an average duration of 9.6 (SD 2.3) minutes. In this group, 95% (19/20) achieved the target HRV (eg, high HRV coherence) for 50% or more of session time in at least 1 completed session. During the postoperative (eg, inpatient stay) period, 70% (16/23) participants completed at ≥1 session (median 2, IQR 1-2.5) with an average duration of 9.5 (SD 2.3) minutes; of which 81% (13/16) successfully achieved the target parameters. Following hospital discharge, 43% (10/23) participants opted to continue VR-BF therapy at home and completed ≥1 session (median 2.5, IQR 2-4) lasting on average 9.2 (SD 1.9) minutes; 80% (8/10) participants were able to achieve the target HRV (Figure 2). More than half of the total participants declined further participation in the study following hospital discharge as they considered Mindfulness Aurora to be “boring.”

Overall, 91% (21/23) of participants completed a session throughout the observational period combined. Of these, 95% (20/21) achieved the target HRV for 50% or more of session time in at least 1 completed session. During the interviews, participants shared that they did not find the contents of Mindfulness Aurora engaging or entertaining enough for them to undergo daily sessions, resulting in reduced completion of postoperative sessions and a small number of patients continuing home use following discharge.

**Table 1:** Participant demographics and medical data.

Variable			Value
Total number of participants, N			23
Age (years), mean (SD)			15.5 (1.8)
Length of hospital stay (nights), mean (SD)			3.1 (4.4)
**Sex, n (%)**			
	Male	7 (30)	
	Female	16 (70)	
**Race, n (%)**			
	African American or Black	3 (13)	
	White	19 (83)	
	Asian	1 (4)	
**Ethnicity, n (%)**			
	Hispanic	1 (4)	
	Non-Hispanic	22 (96)	
**Surgery type, n (%)**			
	Abdominal	3 (13)	
	Bariatric	2 (9)	
	Chest	2 (9)	
	Colorectal	3 (13)	
	Orthopedic	12 (52)	
	Urology	1 (4)	
**ASA^a^ status, n (%)**			
	I or II (healthy or mild systemic disease)	14 (61)	
	III or IV (severe or life-threatening disease)	9 (39)	

^a^ASA: American Society of Anesthesiologists.

### Biofeedback-Based VR Dosing

#### Number of Sessions

The Wilcoxon rank-sum test showed that the number of sessions completed by patients who achieved target HRV (median 4, IQR 2-6) was significantly higher than those who did not reach the target HRV in any of the sessions (median 1.5, IQR 1-2, *P*=.003; Table 2).

**Table 2:** Median (IQR) average duration and the number of biofeedback-based virtual reality sessions of patients completing one or more sessions with 50% or more of session time under target heart rate variability coherence versus those that did not (miss). The Wilcoxon rank-sum test was used to compare the 2 groups.

Patient level outcome			Median (IQR)		*P* value	
**Number of sessions**						.003
	Miss	1.5 (1-2)				
	Achieve target HRV^a^	4 (2-6)				
**Average session duration (minutes)**						.55
	Miss	9.5 (5-11.3)				
	Achieve target HRV	10 (8.6-10.7)				

^a^HRV, heart rate variability

**Figure 3:**
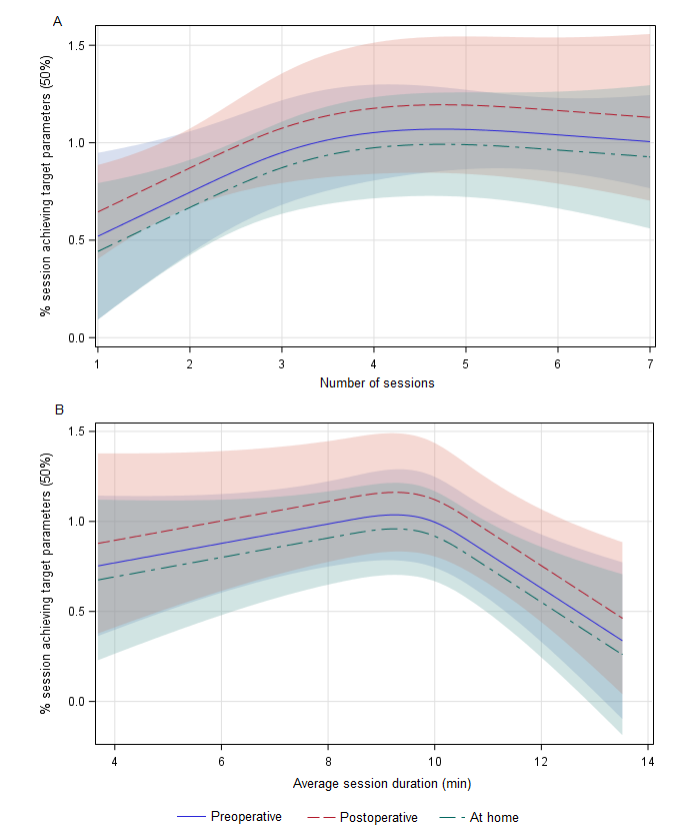
Spline fit of (A) number of sessions and (B) percentage of sessions achieving target parameters.

Nonlinear logistic regression analysis of patient-level outcomes of any session achieving target parameters adjusted for the duration when the perioperative period the sessions were completed showed that participants who completed 4 sessions had the highest odds of having at least 1 session achieving target parameters (odds ratio [OR] 5.1 for 4 vs 3 sessions, 95% CI 1.3-20.6; OR 16.6 for 3 vs 2 sessions, 95% CI 1.2-217.0; Tables 3 and 4). A nonlinear relationship was observed between the patient-level number of sessions and the percentage of sessions resulting in target outcomes. The percentage of sessions resulting in target outcomes increased, then peaked between 4 and 6 sessions (*P*=.04; Figure 3A). However, session-level analysis using nonlinear logistic regression with the outcome of a session achieving target HRV parameters and adjustment for preoperative, postoperative, or home sessions did not show significant associations between session number and achieving target parameters (Table 5). In all analyses, sessions occurring in the preoperative, postoperative, or home periods did not impact any outcomes at either the patient or session level (results not shown).

**Table 3:** Patient-level analysis of nonlinear regression with spline for the number of sessions and average session duration with adjustments for preoperative, postoperative, or home sessions. Logistic regression with outcome—patients achieving target heart rate variability parameters in at least one session.

Session			Odds ratio (95% CI^a^)
**Number of sessions**			
	3 vs 2	16.43 (1.24-217.00)	
	4 vs 3	5.13 (1.28-20.62)	
	5 vs 4	1.89 (0.49-7.34)	
	6 vs 5	1.15 (0.19-6.79)	
**Average session duration**			
	8 vs 7 minutes	1.30 (0.68-2.48)	
	9 vs 8 minutes	1.25 (0.68-2.29)	
	10 vs 9 minutes	0.75 (0.45-1.25)	

^a^CI: Wald CI.

**Table 4:** Patient-level analysis of nonlinear regression with spline for the number of sessions and average session duration with adjustments for preoperative, postoperative, or home sessions. Regression with outcome—percentage of sessions achieving target heart rate variability parameters.

Effect		Least square mean (95% CI^a^)
**Number of sessions**		
	3	0.49 (–0.15 to 1.12)
	4	0.59 (–0.08 to 1.25)
	5	0.60 (–0.08 to 1.28)
	6	0.58 (–0.12 to 1.28)
**Average session duration**		
	7 minutes	0.21 (–0.22 to 0.63)
	8 minutes	0.26 (–0.17 to 0.69)
	9 minutes	0.31 (–0.13 to 0.76)
	10 minutes	0.27 (–0.19 to 0.73)
	11 minutes	0.10 (–0.38 to 0.58)

^a^CI: Wald CI.

**Table 5:** Session-level analysis of nonlinear regression with spline for the number of sessions and average session duration with adjustments for preoperative, postoperative, or home sessions. Logistic regression with outcome—session achieving target heart rate variability parameters.

Session		Odds ratio (95% CI^a^)
**Session number**		
	3 vs 2	1.33 (0.88-2.02)
	4 vs 3	0.81 (0.57-1.15)
	5 vs 4	0.72 (0.46-1.13)
	6 vs 5	0.72 (0.46-1.13)
**Session duration (minutes)**		
	8 vs 7	1.41 (1.09-1.81)
	9 vs 8	1.35 (1.07-1.70)
	10 vs 9	1.04 (0.90-1.21)
	11 vs 10	0.65 (0.51-0.82)
	12 vs 11	0.52 (0.37-0.73)

^a^CI: Wald CI.

#### Session Duration

The Wilcoxon rank-sum test between patients achieving target HRV (median 10, IQR 8.6-10.7 min) versus those that did not (median 9.5, IQR 5-11.3 min) showed that regardless of when the sessions occurred, the average session duration was not associated with HRV concordance (*P*=.55; Table 2).

Nonlinear logistic regression of patient-level outcomes from any session achieving target parameters with adjustment for when the sessions were completed in the perioperative period showed the average session duration did not impact target HRV achievement (Tables 3 and 4). A nonlinear relationship existed between the average session duration and the percentage of sessions resulting in target outcomes at the patient level. The percentage of sessions resulting in target outcomes increased and peaked between an average session duration of 9-10 minutes (*P*=.01; Figure 3B).

Session-level analysis using nonlinear logistic regression with the outcome of a session achieving target HRV parameters and adjustment for preoperative, postoperative, or home sessions showed that session duration is nonlinearly associated with the outcome (spline of session duration *P*<.001) and sessions with a duration of 9 or 10 minutes had the highest odds of achieving target parameters (OR 1.3 for 9 vs 8 min, 95% CI 1.1-1.7; OR 1.4 for 8 vs 7 min, 95% CI 1.1-1.8; OR 1 for 10 vs 9 min, 95% CI 0.9-1.2; Table 5).

### Patient Experience

The PEQ-C and PEQ-P were completed by 87% (20/23) patients and 83% (19/23) parents. Overall, patients and parents both expressed high satisfaction with VR-BF, reporting that they “would recommend VR therapy to friends and family” (15/20, 75% patients; 12/19, 63% parents) and “would use VR again” (12/20, 60% patients; 11/19, 58% parents) if given the opportunity. Patients reported that they “received good preoperative instructions” (18/20, 90%), they “understood how to use the devices” (20/20, 100%), and “the VR technology was easy to use” (19/20, 95%). Similarly, parents agreed that their child “received good instructions” (18/19, 95%) and “could easily use the technology” (15/19, 79%). Few patients “wished the VR experience was more realistic” (8/20, 40%).

During the postoperative period, many patients were “happy to have tried the VR therapy” (16/20, 80%). However, 10% (2/20) of patients reported having “experienced adverse side effects from the VR-BF sessions.” The interviews revealed that 1 participant experienced a mild headache during a session late at night. The second participant reported experiencing nausea due to not wearing prescription glasses during the session. From the parents’ perspectives, nearly half reported that “VR therapy helped them feel better about managing their child’s pain” (9/19, 48%).

Furthermore, 85% (17/20) of patients and 63% (12/19) of parents believed “VR-BF helped with stress and feeling calmer.” In terms of pain reduction, patients (8/20, 40%) and parents (6/19, 32%) reported lower levels of perceived efficacy with VR-BF, and only 5% (1/20) of patients and 11% (2/19) of parents “believed VR-BF helped to reduce consumption of pain medications.” However, the majority of patient (16/20, 80%) and parent (13/19, 68%) responses were neutral on whether they “believed something other than VR-BF would have made the participant feel better.”

## Discussion

This pilot study aimed to develop a future protocol for perioperative VR-BF use in children and adolescents undergoing surgery. Because this technology is novel, assessing its feasibility and acceptability and creating a treatment protocol are essential before designing an efficacy trial. The findings from this work provide preliminary support for the feasibility and acceptability of a perioperative VR-BF intervention for children and adolescents and lay the foundation for the next step in the work to assess the ability of this technology to reduce pain and anxiety in children and adolescents undergoing surgery.

Quantitative results indicated that independent of when sessions occurred, completing between 4 and 6 VR sessions of 9 and 10 minutes was most significantly associated with patients achieving and maintaining target physiological parameters in at least 1 session [27,29]. Qualitative results revealed that perioperative use of VR-BF was well-received by patients and families, particularly in terms of an increase in patient calmness. These findings align with previous literature indicating that mind-body therapies delivered through a gamified virtual world may be viable options for managing pain and anxiety [58]. However, our work also emphasizes the need to tailor content to children’s interests. Because of the lack of variety and type of content (eg, guided relaxation), many patients considered the VR intervention to be boring. As a result, their engagement with the intervention was not as high, which can give rise to a lack of compliance with the intervention [50].

In addition to establishing key parameters for protocol development, the results of the study also identified a number of directions for future research. Due to limited data points from the postoperative periods, the adjusted analysis combined all sessions during the pre-, post-, and home periods. Patients may have been less likely to feel motivated to complete postoperative sessions as many participants reported experiencing elevated pain and stress after surgery, often for the first time, and this may provide insight to why most completed sessions were observed during the preoperative period. Additional research is necessary to determine optimal dosing for postoperative VR-BF application, which may support a different frequency and duration [59]. In addition, participants did not find VR-BF effective for pain reduction, with daily once postoperative use. It is possible that “dosing” VR-BF 3 times daily after surgery could strike a balance between enough uses to achieve target physiologic parameters yet not too onerous to decrease adherence. This regimen is consistent with a study reporting significant transient decreases in pain among hospitalized patients (18 years) using VR versus in-room televisual relaxation programs, a standard of care for all patients [43]. Redesigning the protocol that instructs patients to complete 3 sessions per day after surgery will allow for evaluation of the impact of this frequency and duration on pain reduction in phase 2.

Mind-body therapies, such as yoga, meditation, acupuncture, and even hypnosis, are widely used for chronic pain management. [60]. Only recently, mind-body therapies, including biofeedback, and their relevance to treating acute pain in the perioperative setting have been empirically studied. However, there is insufficient information to establish parameters for efficient HRV-focused biofeedback treatment protocols regarding breathing duration, inhalation/exhalation ratio, body position, or breathing control [61]. A systematic review analyzed protocols implementing HRV-focused biofeedback in 143 studies from the last 20 years and found that many sessions lasted 20 minutes for adults [62], in contrast to 9-10 minutes in our study. Especially in a pediatric population, a shorter session length may be preferred, given the possibility of low motivation for session repetition [50,63] and that younger patients may display an intrinsic apt to master and achieve HRV coherence more quickly and by the first training session in comparison to older patients [64].

To our knowledge, few studies have investigated daily biofeedback use, and even fewer have tried to systematically integrate such interventions into perioperative acute pain care in children and adolescents. In addition, 1 study examined the potential for HRV biofeedback to support self-regulation training in 4 adolescents participating in a chronic pain rehabilitation program and demonstrated improved cardiopulmonary functioning during active training without active feedback, suggesting self-regulation [32]. These results are promising for using HRV biofeedback for children with chronic pain. Still, it requires more extensive research studies with more rigorous methodologies and detailed protocols to support the benefits and implementation of HRV biofeedback in children and adolescents.

With the increasing use of HRV biofeedback therapies in adults, companies like HeartMath have begun developing noninvasive devices to measure HRV for calmness and meditation across the lifespan. This has allowed some critical differences between adults and younger patients to emerge. A pilot study of patients (13-55 years) with eating disorders using the same HeartMath technology as in this study found that younger patients were better at achieving 100% HRV coherence by the first session than older patients [64]. Another study investigating biofeedback and relaxation in children (8-14 years) receiving chemotherapy treatment found significant improvements in HRV coherence by the third and fourth 60-minute sessions [65]. Treatment protocols investigating HRV-focused biofeedback in children and adolescents have ranged from 3 to 36 sessions, each lasting as little as 3 minutes to up to 1 hour [66]. Although there has not been a consensus on the optimal protocol for HRV-focused biofeedback, there is growing acceptance of this therapy, applied independently or as an adjunct to other conventional treatments, in routine medicine for pre- and postsurgical care [24-26].

The immersive environment and sense of awareness created by VR technology are thought to improve patient’s motivation and adherence to a treatment protocol [67], enhancing the therapeutic benefits of complementary medicine. Our previous pilot work using distraction-based VR [49] and guided relaxation-based VR [45] on postoperative pain and anxiety paved the way for combining biofeedback with VR to treat pediatric postoperative pain in a novel and innovative integration of therapies. Distraction-based VR redirects patients’ attention away from the source of their pain. However, without VR, distraction alone yields minimal benefits without any lasting or significant impact on pain relief [42,44,68]. Guided relaxation-based VR, similar to VR-BF, teaches patients relaxation techniques like slow breathing and mindfulness, which can engage parasympathetic or vagal responses to decrease pain [46]. Unlike VR-BF, neither provides patients with instantaneous feedback nor teaches them pain-reducing strategies. A VR-based delivery method may effectively overcome challenges that often hinder the widespread dissemination of conventional mind-body therapies, particularly biofeedback [50]. VR-BF provides an affordable and engaging nonpharmacologic means to safely reduce pain for longer than a brief VR session. Furthermore, as a self-directed tool, VR-BF can potentially reach more patients than biofeedback interventions that rely on clinical instructions and specially trained personnels. The combination of an effective pain and anxiety-reducing tool like biofeedback combined with an immersive technology like VR has the potential to be a very powerful, engaging, and efficacious novel therapy that could be particularly well suited to children and adolescents.

This study has some limitations. The study design prioritized feasibility and acceptability outcomes; therefore, it was not designed as a randomized clinical trial with a control group. A power calculation was not conducted before patient enrollment, and the sample size was established based on the work of previous pilot studies. In addition, the effects on pain, anxiety, and opioid consumption were not measured as this was outside of the scope of this work. The largest limitation with this work was that Mindfulness Aurora and HeartMath do not provide immediate feedback to patients while they are undergoing the VR-BF experience. While the VR game provides a voice-guided narrative to patients telling them how to breathe, and HeartMath captures HRV parameter accomplishment; patients cannot modify or alter their breathing during the experience as they do not receive feedback from the system to help with these modifications. Ultimately, a true VR-BF system would best optimize training in and use of biofeedback in the perioperative period. Having real-time physiological feedback is likely essential to biofeedback learning and will guide patients to improve their performance while progressing through VR-BF sessions.

In summary, this study guided protocol development for the use of VR-BF in the perioperative setting in children and adolescents undergoing surgery. Critically, we found that preoperative VR-BF training that incorporated between 4 and 6 once-per-day sessions, each with a duration of 9-10 minutes, was associated with the highest probability of achievement of target HRV parameters. To enhance protocol adherence and increase the perception of VR-BF as an intervention in the postoperative period, in the next phase of the study, patients will be instructed to complete three 10-minute VR-BF sessions for a total of 7 days after surgery. Our future research plan is to conduct a randomized control trial using the developed protocol [51] to investigate the efficacy of VR-BF in reducing postoperative pain, anxiety, and opioid consumption. Ultimately, this study is essential to developing a nonopioid pain management base in children and adolescents experiencing pain and anxiety.

## Appendix

Multimedia Appendix 1 Patient experience questionnaire – Child (PEQ-C).

Multimedia Appendix 2 Patient experience questionnaire – Parent (PEQ-P).

## Abbreviations

ASA: American Society of Anesthesiologists
CONSORT: Consolidated Standards of Reporting Trials
HRV: heart rate variability
NCH: Nationwide Children’s Hospital
OR: odds ratio
PEQ-C: patient experience questionnaire—child
PEQ-P: patient experience questionnaire—parent
REDCap: Research Electronic Data Capture
VR: virtual reality
VR-BF: biofeedback-based virtual reality

## References

[1] Rabbitts JA, Kain Z (2019). Perioperative care for adolescents undergoing major surgery: a biopsychosocial conceptual framework. Anesth Analg.

[2] Rabbitts JA, Fisher E, Rosenbloom BN, Palermo TM (2017). Prevalence and predictors of chronic postsurgical pain in children: a systematic review and meta-analysis. J Pain.

[3] Fortier MA, Chou J, Maurer EL, Kain ZN (2011). Acute to chronic postoperative pain in children: preliminary findings. J Pediatr Surg.

[4] Rabbitts JA, Zhou C, Groenewald CB, Durkin L, Palermo TM (2015). Trajectories of postsurgical pain in children: risk factors and impact of late pain recovery on long-term health outcomes after major surgery. Pain.

[5] Gan TJ (2017). Poorly controlled postoperative pain: prevalence, consequences, and prevention. J Pain Res.

[6] Peters ML, Sommer M, de Rijke JM, Kessels F, Heineman E, Patijn J, Marcus MAE, Vlaeyen JWS, van Kleef M (2007). Somatic and psychologic predictors of long-term unfavorable outcome after surgical intervention. Ann Surg.

[7] Harbaugh CM, Lee JS, Hu HM, McCabe SE, Voepel-Lewis T, Englesbe MJ, Brummett CM, Waljee JF (2018). Persistent opioid use among pediatric patients after surgery. Pediatrics.

[8] Chambers RA, Taylor JR, Potenza MN (2003). Developmental neurocircuitry of motivation in adolescence: a critical period of addiction vulnerability. Am J Psychiatry.

[9] Groenewald CB, Rabbitts JA, Schroeder DR, Harrison TE (2012). Prevalence of moderate-severe pain in hospitalized children: pain prevalence in hospitalized children. Paediatr Anaesth.

[10] Kozlowski LJ, Kost-Byerly S, Colantuoni E, Thompson CB, Vasquenza KJ, Rothman SK, Billett C, White ED, Yaster M, Monitto CL (2014). Pain prevalence, intensity, assessment and management in a hospitalized pediatric population. Pain Manag Nurs.

[11] Cravero JP, Agarwal R, Berde C, Birmingham P, Coté CJ, Galinkin J, Isaac L, Kost-Byerly S, Krodel D, Maxwell L, Voepel-Lewis T, Sethna N, Wilder R (2019). The society for pediatric anesthesia recommendations for the use of opioids in children during the perioperative period. Paediatr Anaesth.

[12] Hudgins JD, Porter JJ, Monuteaux MC, Bourgeois FT (2019). Trends in opioid prescribing for adolescents and young adults in ambulatory care settings. Pediatrics.

[13] Fisher E, Law E, Palermo TM, Eccleston C (2015). Psychological therapies (remotely delivered) for the management of chronic and recurrent pain in children and adolescents. Cochrane Database Syst Rev.

[14] Wren A, Ross A, D'Souza G, Almgren C, Feinstein A, Marshall A, Golianu B (2019). Multidisciplinary pain management for pediatric patients with acute and chronic pain: a foundational treatment approach when prescribing opioids. Children (Basel).

[15] Keefe FJ, Dunsmore J, Burnett R (1992). Behavioral and cognitive-behavioral approaches to chronic pain: recent advances and future directions. J Consult Clin Psychol.

[16] Zeidan F, Gordon NS, Merchant J, Goolkasian P (2010). The effects of brief mindfulness meditation training on experimentally induced pain. J Pain.

[17] Okifuji A, Ackerlind S (2007). Behavioral medicine approaches to pain. Anesthesiol Clin.

[18] Stern MJ, Guiles RAF, Gevirtz R (2014). HRV biofeedback for pediatric irritable bowel syndrome and functional abdominal pain: a clinical replication series. Appl Psychophysiol Biofeedback.

[19] Pop-Jordanova N (2000). Psychological characteristics and biofeedback mitigation in preadolescents with eating disorders. Pediatr Int.

[20] Gilliland R, Heymen S, Altomare D, Vickers D, Wexner S (1997). Biofeedback for intractable rectal pain: outcome and predictors of success. Dis Colon Rectum.

[21] Heymen S, Wexner SD, Vickers D, Nogueras JJ, Weiss EG, Pikarsky AJ (1999). Prospective, randomized trial comparing four biofeedback techniques for patients with constipation. Dis Colon Rectum.

[22] Hershey AD (2005). Pediatric headache. Pediatr Ann.

[23] Eid M, Aly S, El-Shamy S (2016). Effect of electromyographic biofeedback training on pain, quadriceps muscle strength, and functional ability in juvenile rheumatoid arthritis. Am J Phys Med Rehabil.

[24] Whale K, Wylde V, Beswick A, Rathbone J, Vedhara K, Gooberman-Hill R (2019). Effectiveness and reporting standards of psychological interventions for improving short-term and long-term pain outcomes after total knee replacement: a systematic review. BMJ Open.

[25] Pfeufer D, Gililland J, Böcker W, Kammerlander C, Anderson M, Krähenbühl N, Pelt C (2019). Training with biofeedback devices improves clinical outcome compared to usual care in patients with unilateral TKA: a systematic review. Knee Surg Sports Traumatol Arthrosc.

[26] Mosadeghi S, Reid MW, Martinez B, Rosen BT, Spiegel BMR (2016). Feasibility of an immersive virtual reality intervention for hospitalized patients: an observational cohort study. JMIR Ment Health.

[27] Lehrer PM, Gevirtz R (2014). Heart rate variability biofeedback: how and why does it work?. Front Psychol.

[28] McCraty R, Bradley RT (2009). The coherent heart heart-brain interactions, psychophysiological coherence, and the emergence of system-wide order. Integral Review.

[29] Sowder E, Gevirtz R, Shapiro W, Ebert C (2010). Restoration of vagal tone: a possible mechanism for functional abdominal pain. Appl Psychophysiol Biofeedback.

[30] Zaccaro A, Piarulli A, Laurino M, Garbella E, Menicucci D, Neri B, Gemignani A (2018). How breath-control can change your life: a systematic review on psycho-physiological correlates of slow breathing. Front Hum Neurosci.

[31] Darnall BD, Scheman J, Davin S, Burns JW, Murphy JL, Wilson AC, Kerns RD, Mackey SC (2016). Pain psychology: a global needs assessment and national call to action. Pain Med.

[32] Fahrenkamp A, Benore E (2019). The role of heart rate variability biofeedback in pediatric chronic pain rehabilitation: a case series design. APA.

[33] Malloy KM, Milling LS (2010). The effectiveness of virtual reality distraction for pain reduction: a systematic review. Clin Psychol Rev.

[34] Hoffman HG, Chambers GT, Meyer WJ, Arceneaux LL, Russell WJ, Seibel EJ, Richards TL, Sharar SR, Patterson DR (2011). Virtual reality as an adjunctive non-pharmacologic analgesic for acute burn pain during medical procedures. Ann Behav Med.

[35] Li A, Montaño Zorash, Chen VJ, Gold JI (2011). Virtual reality and pain management: current trends and future directions. Pain Manag.

[36] Garrett B, Taverner T, Masinde W, Gromala D, Shaw C, Negraeff M (2014). A rapid evidence assessment of immersive virtual reality as an adjunct therapy in acute pain management in clinical practice. Clin J Pain.

[37] Gold JI, Kim SH, Kant AJ, Joseph MH, Rizzo AS (2006). Effectiveness of virtual reality for pediatric pain distraction during iv placement. Cyberpsychol Behav.

[38] Carrougher GJ, Hoffman HG, Nakamura D, Lezotte D, Soltani M, Leahy L, Engrav LH, Patterson DR (2009). The effect of virtual reality on pain and range of motion in adults with burn injuries. J Burn Care Res.

[39] Furman E, Jasinevicius TR, Bissada NF, Victoroff KZ, Skillicorn R, Buchner M (2009). Virtual reality distraction for pain control during periodontal scaling and root planing procedures. J Am Dent Assoc.

[40] Morris LD, Louw QA, Crous LC (2010). Feasibility and potential effect of a low-cost virtual reality system on reducing pain and anxiety in adult burn injury patients during physiotherapy in a developing country. Burns.

[41] Indovina P, Barone D, Gallo L, Chirico A, De Pietro G, Giordano A (2018). Virtual reality as a distraction intervention to relieve pain and distress during medical procedures: a comprehensive literature review. Clin J Pain.

[42] Mallari B, Spaeth EK, Goh H, Boyd BS (2019). Virtual reality as an analgesic for acute and chronic pain in adults: a systematic review and meta-analysis. J Pain Res.

[43] Spiegel B, Fuller G, Lopez M, Dupuy T, Noah B, Howard A, Albert M, Tashjian V, Lam R, Ahn J, Dailey F, Rosen BT, Vrahas M, Little M, Garlich J, Dzubur E, IsHak W, Danovitch I (2019). Virtual reality for management of pain in hospitalized patients: a randomized comparative effectiveness trial. PLoS One.

[44] Van Ryckeghem DM, Van Damme S, Eccleston C, Crombez G (2018). The efficacy of attentional distraction and sensory monitoring in chronic pain patients: a meta-analysis. Clin Psychol Rev.

[45] Olbrecht VA, O'Conor KT, Williams SE, Boehmer CO, Marchant GW, Glynn SM, Geisler KJ, Ding L, Yang G, King CD (2021). Guided relaxation-based virtual reality for acute postoperative pain and anxiety in a pediatric population: pilot observational study. J Med Internet Res.

[46] Vagnoli L, Bettini A, Amore E, De Masi S, Messeri A (2019). Relaxation-guided imagery reduces perioperative anxiety and pain in children: a randomized study. Eur J Pediatr.

[47] Mosso JL, Rizzo S, Wiederhold B, Lara V, Flores J, Espiritusanto E, Minor A, Santander A, Avila O, Balice O, Benavides B (2007). Cybertherapy--new applications for discomfort reductions. Surgical care unit of heart, neonatology care unit, transplant kidney care unit, delivery room-cesarean surgery and ambulatory surgery, 27 case reports. Stud Health Technol Inform.

[48] Mosso-Vázquez JL, Gao K, Wiederhold BK, Wiederhold MD (2014). Virtual reality for pain management in cardiac surgery. Cyberpsychol Behav Soc Netw.

[49] Olbrecht VA, O'Conor KT, Williams SE, Boehmer CO, Marchant GW, Glynn SM, Geisler KJ, Pickerill HM, Ding L, Yang G, King CD (2021). Transient reductions in postoperative pain and anxiety with the use of virtual reality in children. Pain Med.

[50] Darnall BD, Krishnamurthy P, Tsuei J, Minor JD (2020). Self-administered skills-based virtual reality intervention for chronic pain: randomized controlled pilot study. JMIR Form Res.

[51] Orgil Z, Johnson L, Karthic A, Williams SE, Ding L, Kashikar-Zuck S, King CD, Olbrecht VA (2023). Feasibility and acceptability of perioperative application of biofeedback-based virtual reality versus active control for pain and anxiety in children and adolescents undergoing surgery: protocol for a pilot randomised controlled trial. BMJ Open.

[52] Prinsloo GE, Derman WE, Lambert MI, Laurie Rauch HG (2013). The effect of a single session of short duration biofeedback-induced deep breathing on measures of heart rate variability during laboratory-induced cognitive stress: a pilot study. Appl Psychophysiol Biofeedback.

[53] ASA Physical Status Classification System.

[54] O'Conor K, Olbrecht VA (2021). Using guided-relaxation based virtual reality to manage post-surgical pain and anxiety in children. Society for Pediatric Pain Medicine Newsletter.

[55] Logan DE, Simons LE, Caruso TJ, Gold JI, Greenleaf W, Griffin A, King CD, Menendez M, Olbrecht VA, Rodriguez S, Silvia M, Stinson JN, Wang E, Williams SE, Wilson L (2021). Leveraging virtual reality and augmented reality to combat chronic pain in youth: position paper from the interdisciplinary network on virtual and augmented technologies for pain management. J Med Internet Res.

[56] Chan A, Tetzlaff JM, Gøtzsche PC, Altman DG, Mann H, Berlin JA, Dickersin K, Hróbjartsson A, Schulz KF, Parulekar WR, Krleza-Jeric K, Laupacis A, Moher D (2013). SPIRIT 2013 explanation and elaboration: guidance for protocols of clinical trials. BMJ.

[57] Craig P, Dieppe P, Macintyre S, Michie S, Nazareth I, Petticrew M (2013). Developing and evaluating complex interventions: the new Medical Research Council guidance. Int J Nurs Stud.

[58] van RM, Lobel A, Harris O, Smit N, Granic I (2016). DEEP: a biofeedback virtual reality game for children at-risk for anxiety.

[59] Coleman CI, Limone B, Sobieraj DM, Lee S, Roberts MS, Kaur R, Alam T (2012). Dosing frequency and medication adherence in chronic disease. J Manag Care Pharm.

[60] Brown ML, Rojas E, Gouda S (2017). A mind-body approach to pediatric pain management. Children (Basel).

[61] Hargett J, Criswell A, Palokas M (2022). Nonpharmacological interventions for acute pain management in patients with opioid abuse or opioid tolerance: a scoping review. JBI Evid Synth.

[62] Lalanza JF, Lorente S, Bullich R, García Carlos, Losilla J, Capdevila L (2023). Methods for heart rate variability biofeedback (HRVB): a systematic review and guidelines. Appl Psychophysiol Biofeedback.

[63] Thorn BE, Pence LB, Ward LC, Kilgo G, Clements KL, Cross TH, Davis AM, Tsui PW (2007). A randomized clinical trial of targeted cognitive behavioral treatment to reduce catastrophizing in chronic headache sufferers. J Pain.

[64] Scolnick B, Mostofsky DI, Keane RJ (2014). Pilot study employing heart rate variability biofeedback training to decrease anxiety in patients with eating disorders. J Eat Disord.

[65] Shockey DP, Menzies V, Glick DF, Taylor AG, Boitnott A, Rovnyak V (2013). Preprocedural distress in children with cancer: an intervention using biofeedback and relaxation. J Pediatr Oncol Nurs.

[66] Dormal V, Vermeulen N, Mejias S (2021). Is heart rate variability biofeedback useful in children and adolescents? A systematic review. J Child Psychol Psychiatry.

[67] Navarro-Haro MV, Modrego-Alarcón M, Hoffman HG, López-Montoyo A, Navarro-Gil M, Montero-Marin J, García-Palacios A, Borao L, García-Campayo J (2019). Evaluation of a mindfulness-based intervention with and without virtual reality dialectical behavior therapy mindfulness skills training for the treatment of generalized anxiety disorder in primary care: a pilot study. Front Psychol.

[68] Eccleston C (2018). Chronic pain as embodied defence: implications for current and future psychological treatments. Pain.

