# Supplementary material for: Use of Biofeedback-Based Virtual Reality in Pediatric Perioperative and Postoperative Settings: Observational Study
Source: JMIR Perioper Med. 2024 Sep 16;7:e48959. doi: 10.2196/48959 (PMC11444093; doi:10.2196/48959)
Supplement: Multimedia Appendix 1 [file periop_v7i1e48959_app1.doc]

Patient Experience Questionnaire - Child (PEQ-C)

Please mark the extent to which you agree or disagree with the following statements:

|  |  | **Strongly Agree** | **Agree** | **Neither Agree nor Disagree** | **Disagree** | **Strongly Disagree** |
| --- | --- | --- | --- | --- | --- | --- |
| **1)** | Before I began virtual reality therapy, I was excited to try it out. | O | O | O | O | O |
| **2)** | After using virtual reality therapy, I was happy that I tried it. | O | O | O | O | O |
| **3)** | Virtual reality therapy helped reduce my pain. | O | O | O | O | O |
| **4)** | Virtual reality therapy helped make me calm. | O | O | O | O | O |
| **5)** | When I use virtual reality therapy, I don’t need as much pain medication. | O | O | O | O | O |
| **6)** | Virtual reality therapy was too much work or too hard. | O | O | O | O | O |
| **7)** | Virtual reality therapy made me dizzy or made my belly upset. | O | O | O | O | O |
| **8)** | I received good instructions before using the device. | O | O | O | O | O |
| **9)** | I understood how to use the device. | O | O | O | O | O |
| **10)** | The virtual reality device was easy to use. | O | O | O | O | O |
| **11)** | The virtual reality device had technical problems. | O | O | O | O | O |
| **12)** | The virtual reality experience felt real. | O | O | O | O | O |
| **13)** | I wish the virtual reality experience was more realistic. | O | O | O | O | O |
| **14)** | I would recommend virtual reality to friends or family. | O | O | O | O | O |
| **15)** | I would use virtual reality again. | O | O | O | O | O |
| **16)** | I would rather try virtual reality to help my pain than take pain medication. | O | O | O | O | O |
| **17)** | Something other than virtual reality would have made me feel better. | O | O | O | O | O |
| **18)** | I wish I had not received virtual reality therapy. | O | O | O | O | O |
| **19)** | I was already familiar with virtual reality technology. | O | O | O | O | O |
|  |  |  |  |  |  |  |
