# Supplementary material for: Use of Biofeedback-Based Virtual Reality in Pediatric Perioperative and Postoperative Settings: Observational Study
Source: JMIR Perioper Med. 2024 Sep 16;7:e48959. doi: 10.2196/48959 (PMC11444093; doi:10.2196/48959)
Supplement: Multimedia Appendix 2 [file periop_v7i1e48959_app2.doc]

Patient Experience Questionnaire - Parent (PEQ-P)

Please mark the extent to which you agree or disagree with the following statements:

|  |  | **Strongly Agree** | **Agree** | **Neither Agree nor Disagree** | **Disagree** | **Strongly Disagree** |
| --- | --- | --- | --- | --- | --- | --- |
| **1)** | Before my child began virtual reality therapy, he/she was excited to try it out. | O | O | O | O | O |
| **2)** | After my child used virtual reality therapy, he/she was happy that he/she had tried it. | O | O | O | O | O |
| **3)** | Virtual reality therapy helped reduce my child’s pain. | O | O | O | O | O |
| **4)** | Virtual reality therapy helped make my child calm. | O | O | O | O | O |
| **5)** | When my child uses virtual reality therapy, he/she doesn’t need as much pain medication. | O | O | O | O | O |
| **6)** | Virtual reality therapy was too much work or too hard for my child. | O | O | O | O | O |
| **7)** | Virtual reality therapy made my child dizzy or nauseous. | O | O | O | O | O |
| **8)** | My child received good instructions before using the device. | O | O | O | O | O |
| **9)** | My child understood how to use to device. | O | O | O | O | O |
| **10)** | The virtual reality device was easy for my child to use. | O | O | O | O | O |
| **11)** | The virtual reality device had technical problems. | O | O | O | O | O |
| **12)** | The virtual reality therapy made me feel better about managing my child’s pain. | O | O | O | O | O |
| **13)** | I would recommend virtual reality to friends or family. | O | O | O | O | O |
| **14)** | I would want my child to use virtual reality again. | O | O | O | O | O |
| **15)** | I would prefer that my child use virtual reality than take medications to reduce his/her pain. | O | O | O | O | O |
| **16)** | Something other than virtual reality would have made my child feel better. | O | O | O | O | O |
| **17)** | I wish my child had not received virtual reality therapy. | O | O | O | O | O |
| **18)** | I am familiar with virtual reality technology. | O | O | O | O | O |
